# Adult Mental Health Presentations to Emergency Departments in Victoria, Australia between January 2018 and October 2020: Changes Associated with COVID-19 Pandemic Public Health Restrictions

**DOI:** 10.1007/s11126-023-10057-4

**Published:** 2023-11-08

**Authors:** Jackson Newberry-Dupé, Wanyu Chu, Simon Craig, Rohan Borschmann, Gerard O’Reilly, Paul Yates, Glenn Melvin, Kylie King, Harriet Hiscock

**Affiliations:** 1 Centre for Mental Health, Melbourne School of Population and Global Health, University of Melbourne, Melbourne, VIC Australia; 2 Centre for Adolescent Health, Murdoch Children’s Research Institute, Royal Children’s Hospital, 50 Flemington Rd, Parkville, Melbourne, VIC 3052 Australia; 3 Centre for Health Services, Murdoch Children’s Research Institute, Royal Children’s Hospital, Melbourne, VIC Australia; 4 Paediatric Emergency Department, Monash Medical Centre, Emergency Service, Monash Health, Clayton, VIC Australia; 5 Department of Paediatrics, School of Clinical Sciences, Monash University, Clayton, VIC Australia; 6 Emergency Research Group, Murdoch Children’s Research Institute, Parkville, VIC Australia; 7 Melbourne School of Psychological Sciences, University of Melbourne, Parkville, VIC Australia; 8 Department of Psychiatry, Warneford Hospital, University of Oxford, Oxford, OX3 7JX UK; 9 Emergency and Trauma Centre, The Alfred, Melbourne, Australia; 10 National Trauma Research Institute, The Alfred, Melbourne, Australia; 11 School of Public Health and Preventive Medicine, Alfred Campus, Monash University, Melbourne, Australia; 12 Department of Geriatric Medicine, Continuing Care Clinical Service Unit, Austin Health, Heidelberg, Australia; 13 Faculty of Medicine, Dentistry and Health Sciences, University of Melbourne, Melbourne, Australia; 14 School of Psychology, Deakin University, Melbourne, VIC Australia; 15 Turner Institute for Brain and Mental Health, Monash University, Victoria, Australia; 16 Centre for Community Child Health, Royal Children’s Hospital, Melbourne, VIC Australia; 17 Department of Paediatrics, University of Melbourne, Melbourne, Australia

**Keywords:** Mental health, COVID-19, Emergency department, Lockdown, Adult, Victoria, Australia

## Abstract

The COVID-19 pandemic and associated public health measures altered patterns of help-seeking for mental health, with increases in emergency department utilisation reported. We examined the association between COVID-19 restrictions and adult emergency department (ED) mental health presentations in Victoria, Australia, through secondary analysis of data from 39 public EDs across the state. Participants were all patients (18+ years) presenting between 1 January 2018 and 31 October 2020 with mental health or intentional self-harm. The main outcome was number of presentations for each mental health condition, by patient age, socioeconomic status (SES), location, and ED triage category. We used a Poisson regression model to compare predicted monthly ED presentations based on trends from 2018, 2019 and 2020 (up to 31 March), with observed presentations during the initial months of the COVID-19 pandemic (1 April to 31 October 2020). There was an average of 4,967 adult mental health presentations per month pre-COVID-19 (1 January–31 March 2020) and 5,054 per month during the COVID-19 period (1 April–31 October 2020). Compared to predicted incidence, eating disorder presentations increased 24.0% in the COVID-19 period, primarily among higher SES females aged 18–24 years. Developmental/behavioural disorder presentations decreased by 19.7% for all age groups. Pandemic restrictions were associated with overall increases in monthly adult ED presentations for mental health, with some disorders increasing and others decreasing. Accessibility of acute mental health services needs to be addressed to meet changing demand and ensure services are responsive to changes in presentations resulting from future public health challenges.

## Introduction

The implementation of social restrictions by public health authorities was an important tool in combating the novel Coronavirus (COVID-19) pandemic. However, such measures were associated with increased psychological distress [1]. This distress was not distributed evenly within the population, with greater impact on those with lower income, existing mental health problems and/or special needs, minority groups, families, and young people [1, 2].

On 25 January 2020, the first reported cases of COVID-19 in Australia were discovered [3]. In the state of Victoria (population 6.7 million) [4], the rising number of COVID-19 cases prompted implementation of Stage 3 restrictions on 30 March 2020. Only essential businesses were permitted to operate, and outdoor gatherings were restricted to two people. Education facilities were closed, and a stay-at-home order was issued [5]. Metropolitan areas in Victoria were locked down for approximately five months over two stay-at-home periods (30 March to 12 May and 8 July to 27 October) in 2020, the longest and most rigorous restrictions in the country and the strictest in the world at that time [3]. Through analysis of survey data collected between May 2020 and December 2021, Botha and colleagues [6] identified a proportional increase in psychological distress in Australian adults across five states during periods of lockdown, with longer lockdowns associated with greater distress.

Medicare data from the Australian Institute of Health and Welfare (AIHW) have demonstrated that usage of primary healthcare consultations for mental health – the service most frequently accessed by people in Australia – increased during the lockdown periods in Victoria [7]. Usage increased by 4.9% in June 2020 (rate of 4,882 services per 100,000 population) compared to the same time in 2019. The highest demand for services was observed over the four weeks to 13 September 2020, in which 358,909 services were accessed (5,361 services per 100,000 population). Concurrently, an increase in the proportion of primary healthcare consultations being accessed through telehealth was observed, with over 60% of services in August and September delivered by telephone or video conferencing [7]. This may be at least partially attributed to the addition of a wide range of telehealth options to the Medicare Benefits Scheme by the Australian Government over the course of the COVID-19 pandemic [7].

In 2020 during the early months of the pandemic, a decrease in hospital emergency department (ED) presentations for mental health was reported in many countries, corresponding with decreases in other (non-COVID-19-related) presentations [8]. This has been attributed to a combination of fear around hospital outbreaks, hospital overcrowding, service closures, and stay at home orders and pre-emptive restrictions on resident movements in many aged care facilities (including hospitalization leading to reduced access and delayed presentations) [9–11]. While some international studies reported sustained decreases in mental health presentations following this decline [12, 13], others have reported increases among certain demographics (e.g. young people) and for certain diagnoses [14]. There have been mixed results from health-service studies in Victoria Australia, which have investigated high-level data (e.g., “psychiatric presentations”) [15] or specific disorders (e.g., schizophrenia and psychotic disorders) [16], with limited disaggregation of data based on patient demographics, socio-economic status (SES), or metropolitan vs. regional contexts.

There is a need for more nuanced data across multiple sites and over longer collection periods to determine how the restrictions may have been associated with different adult mental health conditions and age groups in both metropolitan and regional contexts. This knowledge could be useful in improving resource use in Victorian hospital EDs, by identifying areas where targeted interventions for emergency mental health access and care can be directed.

In this study we aimed to 1) quantify changes in the number of adult (18+ years) presentations pre- and post-restrictions for common mental health diagnoses to 39 public hospital EDs in Victoria, Australia; and 2) determine whether – and to what extent – changes differed by mental health condition, age, sex, socioeconomic status, and geographic remoteness.

## Method

### Study Design

To assess changes in mental health presentations to Victorian EDs associated with the COVID-19 pandemic, we utilized an interrupted time-series design with multiple cross-sectional observations. Interrupted time-series designs are among the strongest quasi-experimental designs where RCT’s are unable to be conducted, such as in the case of a global pandemic [17]. The manuscript was prepared with reference to the reporting of studies conducted using observational routinely collected health data (RECORD) Statement [18]. The study obtained organisational approval from The Royal Children's Hospital (Melbourne) for an exemption from ethical review (Project ID: 70069).

### Data Source

We obtained data on adult (18+ years) ED presentations from the Victorian Emergency Minimum Dataset (VEMD) for the period 1 January 2018 to 31 October 2020. The VEMD comprises mandatorily collected de-identified demographic, administrative and clinical data detailing ED presentations at all 39 Victorian public hospitals with a designated ED [19]. Demographic data found in the VEMD includes sex (male or female), age, country of birth, Indigenous status, and residential postcode. Within the VEMD, the reasons for ED presentation are coded as the principal diagnosis using a shortened version of the International Statistical Classification of Diseases and Related Health Problems, Tenth Revision, Australian Modification (ICD-10-AM) codes (approximately 1,100 codes compared to over 120,000 in the full index) [20].

### Study Population and Outcomes

Demographic data were obtained on the sex and age of patients included in our analysis. For the purposes of our analysis, age was grouped into three categories: 18 to 24 years, 25 to 64 years, and 65 and older. Mental health presentations were defined as those leading to an F group diagnosis (F00-F99; mental and behavioural disorders) according to ICD-10-AM or a diagnosis of intentional self-harm as identified by ICD-10-AM code X60-X84 as well as S or T codes (injuries and poisonings) with intent of self-harm. Only the primary presenting diagnosis is included in the VEMD, determined upon conclusion of the patient’s ED visit as the main reason for their attendance after clinical assessments have been considered [19]. We analysed trends for eleven common conditions: dementia; delirium; alcohol related disorders; substance abuse; schizophrenia, schizotypal and delusional disorders; mood disorders; anxiety disorders; eating disorders; personality disorders; self-harm; and developmental and behavioural disorders (e.g., autism spectrum disorder, attention deficit/hyperactivity disorder). A full list of ICD-10-AM codes used in our study can be found in Appendix. The main outcome of interest was the number of presentations per month for each condition. The number of patients per month was included as a secondary outcome, to estimate changes in the presentation-to-patient ratio.

ED presentation urgency is defined in the VEMD using the five-level Australasian Triage Scale (ATS) [21]. For the purposes of this analysis, a binary variable has been derived from these five levels to indicate whether the presentation was an urgent (ATS code of l or 2 in VEMD) or non-urgent episode (ATS code of 3–5).

Remoteness is a binary variable derived from patient postcodes in the VEMD, indicating whether the presenting patient’s residential postcode was in regional Victoria or metropolitan Melbourne. Remoteness was defined based on the Australian Bureau of Statistics (ABS) Remoteness Areas Structure within the Australian Statistical Geography Standard (ASGS) [22]. This structure divides Australia into five categories of remoteness based on a measure of relative access to services: major cities (metropolitan Melbourne), inner regional, outer regional, remote, and very remote (regional Victoria). Metropolitan Melbourne is home to 78.1% of the Victorian population [4] and contains 22 of the 39 Victorian public hospitals included in our analysis [23, 24].

Socioeconomic status (SES) is a binary variable derived from patient residential postcodes in the VEMD and the ABS-developed Socio-Economic Indexes for Areas (SEIFA), indicating whether the episode occurred in a lower or higher SES area. SEIFA ranks areas in Australia using postcode according to relative socio-economic advantage and disadvantage. The Index of Relative Socio-economic Disadvantage (IRSD) is one of the four indexes in SEIFA that summarises a range of information about the economic and social conditions of people and households within an area by area-based deciles [25]. Area-based deciles are calculated by dividing the areas, ordered by disadvantage, into 10 equally sized groups. Lower SES included areas that have a decile number of 1–5, while higher SES included areas that have a decile number of 6–10.

### Statistical Analysis

ED episodes were defined as the monthly incidence of ED presentations rather than the number of patients presenting. To examine the change in health service use before and after the COVID-19 pandemic restrictions, the number of ED episodes was converted into a monthly time series format and the entire observational period was divided into a pre-COVID-19 period (1 January 2018 to 31 March 2020) and a COVID-19 period (1 April 2020 to 31 October 2020, in line with the start of the first stage 3 lockdown in Victoria on 31 March 2020 and the end of the second lockdown in metropolitan Melbourne on 28 October 2020) [5]. Thus, the study period comprised 27 “pre-COVID-19” and seven “COVID-19” months of observations, totaling 34 observation points. A ratio of the number of presentations to patients was calculated for the pre-COVID-19 and COVID-19 periods to assess changes in repeat presentations.

We built a Poisson prediction model using the 27 data points occurring in the pre-COVID-19 period, with the number of presentations as the outcome variable and the time (month) since the start of the observational period as the predictor. The model was designed to predict the monthly number of presentations in the COVID-19 period if the COVID-19 pandemic and associated restrictions had not occurred and the pre-COVID-19 trend had continued [26]. To quantify the uncertainty in the randomness associated with the point being predicted as well as in the coefficient estimates, the 95% prediction interval (PI) of the predicted ED presentation number was also reported. The difference between the observed and the predicted frequency was computed. The relative difference was calculated by subtracting the predicted from the observed frequency then dividing by the predicted frequency. Then for each condition, a mean difference was computed by averaging the sum of the monthly differences in the COVID-19 period and a mean relative difference was computed by averaging the sum of the relative differences of each month in the COVID-19 period.

Subgroup analyses by remoteness, socioeconomic status, triage category, admitted proportion, sex, and age were conducted using the chi-square test to investigate any within-subgroup change in the ED presentation rate before and during the COVID period. All analyses were performed in R version 4.0.3 [27].

## Results

**Fig. 1:**
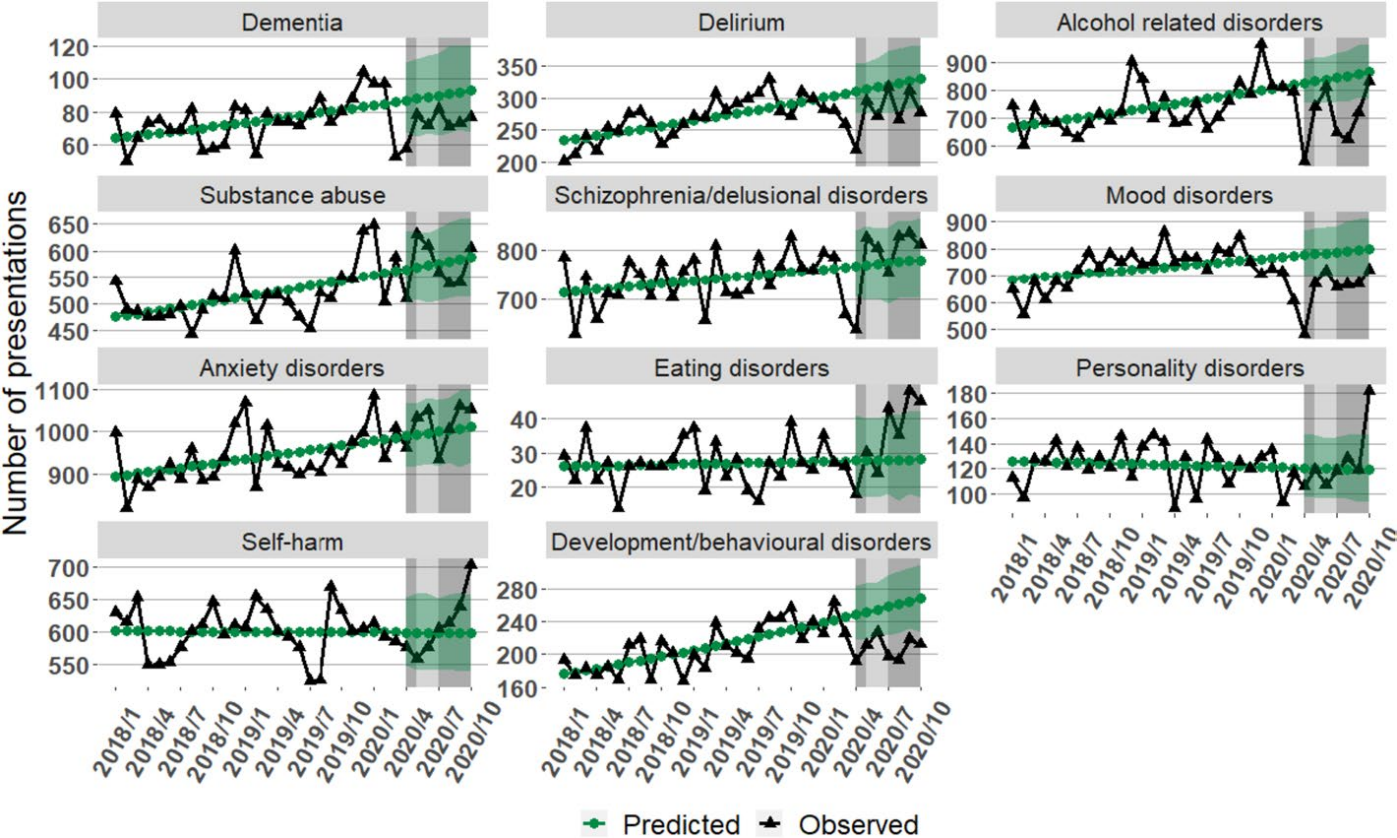
Predicted and observed trends in presentations for common MH conditions to Victorian EDs pre-COVID-19 vs COVID-19 (Shaded grey area: time in 2020 affected by COVID-19; Darker grey area: 1^st^ & 2^nd^ lockdown in metro Victoria; Shaded green area: 95% prediction interval of predicted presentations in the COVID-19 period, based on 2018 and 2019 data)

**Fig. 2:**
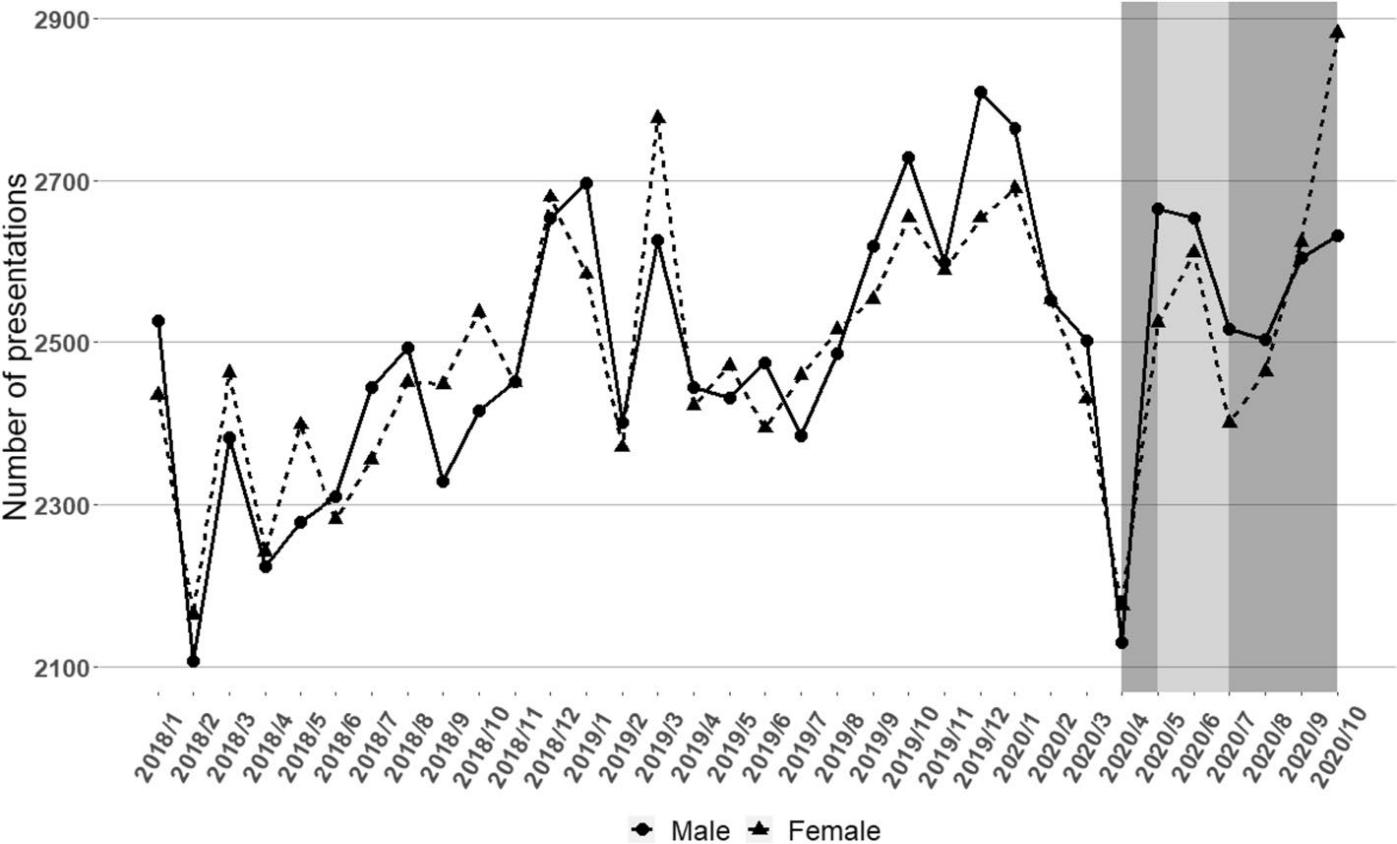
Trends in MH presentations to Victorian EDs from 2018 to 2020, stratified by Sex** (**Shaded grey area: time in 2020 affected by COVID-19; Darker grey area: 1^st^ & 2^nd^ lockdown in metro Victoria)

Table 1 shows the characteristics of presentations for the pre-COVID-19 and COVID-19 periods by condition, whilst Fig. 1 shows the number of predicted and observed presentations over the same period. Figure 2 shows the breakdown of presentations by sex. Overall, there was a reduction in the number of ED presentations for mental health conditions in the month immediately preceding each lockdown, followed by a sharp increase during lockdowns (Figs. 1 and 2). This change was driven by metropolitan Melbourne, with largely stable presentation numbers for mental health conditions in regional Victoria during the study period. The number and pattern of presentations by males and females was similar overall, although the conditions they most frequently presented with differed (Table 1). However, a rapid increase in ED presentation numbers was observed through the second lockdown in women, but not in men (Fig. 2). Most presentations were by adults aged 25–65 years, with a mean age of 41.1 (SD: 18.3) years. There was an increase in presentations with an urgent triage category towards the end of the second lockdown. The proportion of repeat presentations for alcohol related disorders, schizophrenia/delusional disorders, eating disorders, and personality disorders increased from the pre-COVID-19 period to the COVID-19 period.

**Table 1:** Characteristics of MH presentations to Victorian EDs pre-COVID-19 (Jan 2018 to Mar 2020) and during COVID-19 (Apr 2020 to Oct 2020)

	**No. of presentations**			**No. of patients**			**Presentation/patient ratio**		**Age (mean (SD))**	
**Condition**	**Pre-COVID (27 months)**	**COVID (7 months)**	**Total**	**Pre-COVID (27 months)**	**COVID (7 months)**	**Total**	**Pre-COVID (27 months)**	**COVID (7 months)**	**Pre-COVID (27 months)**	**COVID (7 months)**
**Dementia**	2013	511	2524	1817	441	2258	1.1	1.2	81.0 (8.8)	81.1 (8.0)
**Delirium**	7247	1956	9203	6631	1714	8345	1.1	1.1	79.3 (12.7)	79.6 (12.1)
**Alcohol related disorders**	19966	4905	24871	12619	2439	15058	1.6	2	41.9 (15.3)	42.9 (14.4)
**Substance abuse**	13954	3991	17945	10915	2743	13658	1.3	1.5	34.1 (12.2)	33.5 (11.4)
**Schizophrenia/delusional disorders**	19985	5492	25477	12026	2607	14633	1.7	2.1	38.9 (12.8)	39.0 (13.1)
**Mood disorders**	19675	4582	24257	16109	3409	19518	1.2	1.3	38.9 (15.8)	38.5 (16.0)
**Anxiety disorders**	25382	7098	32480	21108	5394	26502	1.2	1.3	40.5 (17.3)	40.2 (17.1)
**Eating disorders**	723	243	966	445	129	574	1.6	1.9	27.7 (12.3)	25.2 (9.1)
**Personality disorders**	3331	879	4210	1977	422	2399	1.7	2.1	32.2 (11.5)	31.2 (11.1)
**Self-harm**	16206	4271	20477	11965	2718	14683	1.4	1.6	35.1 (14.7)	33.9 (14.1)
**Development/behavioural disorders**	5636	1450	7086	4877	1147	6024	1.2	1.3	36.9 (14.3)	36.6 (14.2)

**Fig. 3:**
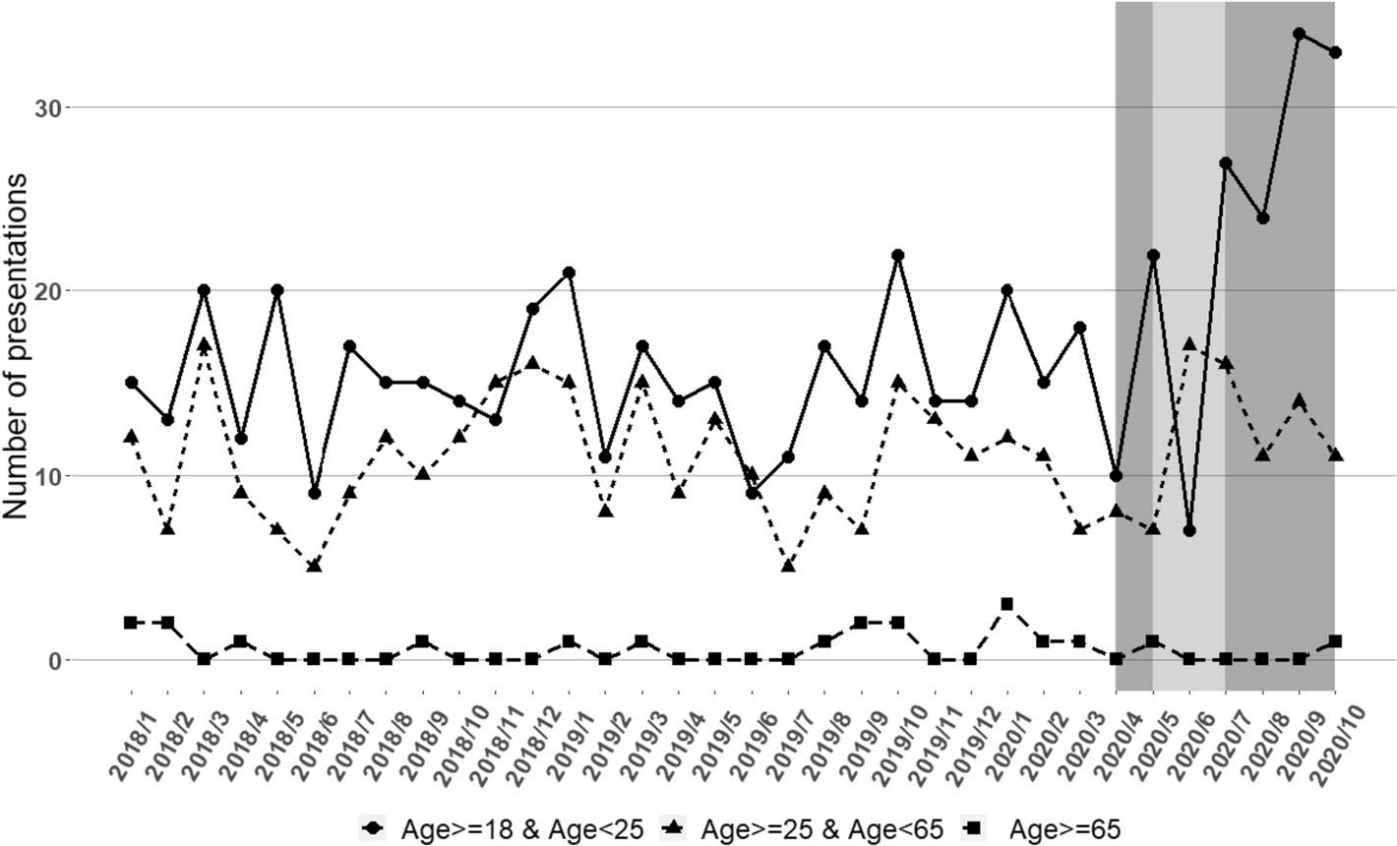
Trends in presentations for eating disorders to Victorian EDs from 2018 to 2020, stratified by age (Shaded grey area: time in 2020 affected by COVID-19; Darker grey area: 1^st^ & 2^nd^ lockdown in metro Victoria)

Table 2 shows the predicted (based on the pre-COVID-19 trends) versus observed number of presentations by mental health condition per month in the COVID-19 period and the relative difference between observed and predicted for all patients aged 18+ years. Between 1 April 2020 and 31 October in 2020, eating disorders had the largest relative difference in presentations overall, with 24% more presentations than predicted; this increase was only observed in adults aged 18–24 years (Fig. 3). Developmental and behavioural disorders had the second largest difference, with 19.7% fewer presentations in 2020 than predicted (Fig. 1; Table 2).

**Table 2:** Observed vs. predicted number of monthly presentations to Victorian EDs for common MH conditions Apr to Oct 2020 (COVID-19)

	**Dementia**				**Delirium**			
	Observed	Predicted (95% PI)	Difference	Relative difference (%)	Observed	Predicted (95% PI)	Difference	Relative difference (%)
Apr-20	58	87 (68, 110)	-29	-33.3	219	310 (275, 353)	-91	-29.4
May-20	78	88 (65, 112)	-10	-11.4	295	313 (276, 355)	-18	-5.8
Jun-20	72	89 (67, 114)	-17	-19.1	271	316 (278, 363)	-45	-14.2
Jul-20	82	90 (66, 116)	-8	-8.9	317	320 (275, 373)	-3	-0.9
Aug-20	71	91 (67, 120)	-20	-22.0	266	323 (280, 376)	-57	-17.6
Sep-20	73	92 (69, 120)	-19	-20.7	312	327 (282, 380)	-15	-4.6
Oct-20	77	93 (68, 121)	-16	-17.2	276	330 (285, 383)	-54	-16.4
Mean			-17	-18.9			-40	-12.7

	**Alcohol related disorders**				**Substance abuse**			
	Observed	Predicted (95% PI)	Difference	Relative difference (%)	Observed	Predicted (95% PI)	Difference	Relative difference (%)
Apr-20	543	824 (751, 908)	-281	-34.1	509	565 (501, 636)	-56	-9.9
May-20	738	830 (754, 917)	-92	-11.1	630	569 (509, 635)	61	10.7
Jun-20	808	837 (751, 919)	-29	-3.5	610	572 (502, 634)	38	6.6
Jul-20	645	843 (755, 932)	-198	-23.5	560	576 (505, 642)	-16	-2.8
Aug-20	623	850 (757, 942)	-227	-26.7	538	580 (511, 653)	-42	-7.2
Sep-20	718	857 (774, 959)	-139	-16.2	539	583 (514, 659)	-44	-7.5
Oct-20	830	864 (767, 963)	-34	-3.9	605	587 (513, 661)	18	3.1
Mean			-143	-17.0			-6	-1.0

	**Schizophrenia/delusional disorders**				**Mood disorders**			
	Observed	Predicted (95% PI)	Difference	Relative difference (%)	Observed	Predicted (95% PI)	Difference	Relative difference (%)
Apr-20	639	768 (708, 839)	-129	-16.8	483	777 (707, 866)	-294	-37.8
May-20	824	770 (700, 848)	54	7.0	670	781 (701, 877)	-111	-14.2
Jun-20	803	772 (704, 844)	31	4.0	715	784 (699, 880)	-69	-8.8
Jul-20	755	774 (692, 846)	-19	-2.5	658	788 (700, 886)	-130	-16.5
Aug-20	828	776 (703, 859)	52	6.7	665	792 (699, 901)	-127	-16.0
Sep-20	833	778 (702, 857)	55	7.1	669	795 (705, 911)	-126	-15.8
Oct-20	810	780 (702, 864)	30	3.8	722	799 (697, 913)	-77	-9.6
Mean			11	1.3			-133	-17.0

	**Anxiety disorders**				**Eating disorders**			
	Observed	Predicted (95% PI)	Difference	Relative difference (%)	Observed	Predicted (95% PI)	Difference	Relative difference (%)
Apr-20	962	990 (916, 1069)	-28	-2.8	18	28 (18, 41)	-10	-35.7
May-20	1034	993 (921, 1067)	41	4.1	30	28 (17, 40)	2	7.1
Jun-20	1050	997 (925, 1079)	53	5.3	24	28 (17, 40)	-4	-14.3
Jul-20	935	1001 (920, 1074)	-66	-6.6	43	28 (18, 41)	15	53.6
Aug-20	1005	1004 (927, 1081)	1	0.1	35	28 (16, 41)	7	25.0
Sep-20	1061	1008 (917, 1096)	53	5.3	48	28 (18, 42)	20	71.4
Oct-20	1051	1012 (928, 1096)	39	3.9	45	28 (17, 42)	17	60.7
Mean			13	1.3			7	24.0

	**Personality disorders**				**Self-harm**			
	Observed	Predicted (95% PI)	Difference	Relative difference (%)	Observed	Predicted (95% PI)	Difference	Relative difference (%)
Apr-20	106	121 (97, 147)	-15	-12.4	576	599 (549, 654)	-23	-3.8
May-20	119	120 (97, 147)	-1	-0.8	559	599 (542, 658)	-40	-6.7
Jun-20	107	120 (96, 144)	-13	-10.8	577	599 (542, 658)	-22	-3.7
Jul-20	118	120 (96, 145)	-2	-1.7	605	599 (541, 651)	6	1.0
Aug-20	128	120 (96, 147)	8	6.7	613	599 (544, 656)	14	2.3
Sep-20	119	120 (94, 147)	-1	-0.8	638	599 (539, 661)	39	6.5
Oct-20	182	119 (94, 147)	63	52.9	703	599 (539, 657)	104	17.4
Mean			6	4.7			11	1.9

**Development/behavioural disorders**			
Observed	Predicted (95% PI)	Difference	Relative difference (%)
192	248 (218, 283)	-56	-22.6
211	251 (219, 287)	-40	-15.9
227	255 (223, 288)	-28	-11.0
197	258 (223, 297)	-61	-23.6
193	261 (226, 301)	-68	-26.1
218	265 (231, 305)	-47	-17.7
212	268 (228, 309)	-56	-20.9
		-51	-19.7

Many mental health presentations reduced substantially during the first lockdown in April before returning to pre-pandemic levels over May–June (Table 2; Fig. 1). Substance abuse presentations also returned in October, but to higher levels than predicted based on pre-pandemic presentations. Eating disorder and personality disorder presentations were elevated, with a more marked increase in the number of eating disorder presentations compared to predicted levels (71.4% more eating disorder presentations than predicted in September, 60.7% higher in October). There were unexpected changes in presentations for alcohol related disorders, dementia, delirium, schizophrenia/delusional disorders, mood disorders, eating disorders, personality disorders, self-harm and developmental/behavioural disorders that fell outside the 95% prediction interval (Fig. 1). Finally, people from lower SES areas were more likely to present to ED for mental health conditions than people from higher SES areas, except for eating disorders where this trend was reversed (Table 1).

## Discussion

Following an initial reduction in mental health presentations before lockdown, our analysis identified a large increase in adult ED presentations to Victorian hospitals for eating disorders in 2020 (based on predictions from previous years) and a reduction in presentations for developmental / behavioural disorders. The increase in eating disorder presentations was driven by young adults (primarily female) in metropolitan Melbourne, predominantly from higher SES backgrounds. Mental health presentations rose sharply for females in Victoria during the second lockdown, but not for males.

The pattern of mental health presentations at the onset of the pandemic and following implementation of lockdowns was similar to those seen in other countries for substance abuse and alcohol related disorders [13], dementia and delirium [28], and self-harm [29]. Presentations dropped markedly in the lead up to lockdowns, followed by a partial or complete return to pre-pandemic levels [13]. The early decrease in presentations may be due to hesitation to attend the ED for fear of infection, a phenomenon that was widely reported globally [9, 10]. However, unlike many countries where this fear continued to impact presentations, there were comparatively few COVID-19 cases in Australia during the study period. This may partially explain the rapid resurgence in presentations for certain mental health conditions shortly after the early decline.

There was evidence of an increase in triage urgency towards the end of the second lockdown, a finding also identified in some international studies (e.g., 29). This may indicate increased severity of acute symptoms for mental health presentations. However, it could also be an artefact of the reduction in presentations observed at the onset of restrictions, where persons with less severe symptoms may have disproportionately chosen not to present to hospital. National data from the AIHW on presentations in 2021 suggests that presentations quickly rebounded to pre-pandemic levels when lockdowns were lifted [30]. This differs from some international findings, with one study of a national database of health contacts in South Korea reporting that ED presentations remained low even after lockdowns had lifted [31]. Further research is needed to determine whether deferred care during lockdowns in 2020 and 2021 resulted in rebounding presentation numbers or increased severity of health conditions in the absence of care.

The rise in mental health presentations by females, but not males, aligns with data from the AIHW, which indicated that in 2020/2021, young women represented the fasted growing demographic presenting to hospitals for mental health reasons, with highest rates of mental health presentations in the 12–17-year age group, followed by women aged 18–24 years [30]. This finding differed from some international results. For example, studies in Türkiye and Switzerland found no significant difference in thesex of patients presenting to ED for mental health reasons before and during the COVID-19 pandemic [29, 32], A community-based study demonstrated heightened depression and anxiety amongst Australian women compared to men during COVID-19 restriction periods, associated with a greater burden of unpaid work caring for children [33]. However, given that only 1.0% of women in Victoria who gave birth in 2020 were under 20 years old [34], this is unlikely to account for the higher rates observed in younger women. Sex differences in help-seeking behaviour [35] and higher job-losses in female dominated industries [36] may be more important contributing factors. Similarly, the increase in presentations by young adults (18–24 years) suggests that lockdowns in Victoria were particularly difficult for younger people, who may have been especially vulnerable to restrictions on social gatherings and hospitality, retail, and tourism businesses.

Contrary to some international findings [29], no significant increase in adult presentations for self-harm and suicidal behaviour was observed during the COVID-19 period, despite increased psychological distress and suicidal ideation in the Victorian community during 2020 [37]. This aligns with evidence that the overall number of suicides did not increase in Victoria in 2020 compared to previous years [38], despite worrying increases in vulnerable groups including Indigenous Australians [39]. The reasons for this are likely to be complex but may in part be attributed to reductions in stressors and risk factors for suicide. For example, the risk of poverty and unemployment was reduced by the availability of JobKeeper and JobSeeker supplements [40]. While this could be a positive outcome for adult suicide prevention, a different story has emerged for young people. Australian children and adolescents aged ≤ 18 years were found to have presented to ED for self-harm and suicidal ideation at higher than predicted rates in Victoria during 2020 [41] and NSW during lockdowns in 2021 [42]. As young people have comparatively higher baseline levels of self-harm and suicidal behaviour than those aged 18 years and over, the increases in this group are particularly concerning [43]. Furthermore, hesitancy to attend the ED for non-life-threatening self-harm during the COVID-19 period may have led to reduced adult presentations despite comparable or higher incidence in the community.

Another factor to consider is access to primary care and community psychological services, use of which increased during the Victorian lockdown periods [40]. This could indicate that reductions in presentations to the ED may be explained by people preferencing primary care over hospitals due to COVID-19 related concerns. However, there were anecdotal reports of psychologists and psychiatrists ceasing to accept new referrals, leaving patients with fewer treatment options [44]. Furthermore, most primary care operators moved to a telehealth or e-health platform during the Victorian lockdowns in 2020. Some patients, particularly older adults with cognitive impairments may have found this difficult to access or unsatisfactory. Older adults faced significant barriers to care, particularly those with cognitive impairment, with many visiting services suspending face-to-face support [45, 46].

Older adults with mental health issues and other comorbidities are at particularly heightened risk of deteriorating health from deferred care [45]. However, there was no evidence of an increase in presentations for older adults with dementia and delirium during our study period, despite reports of increased vulnerability and limited access to community health and support services [45, 46]. Several factors may help to explain this finding. Firstly, VEMD only captures the primary presenting diagnosis, meaning that mental health conditions such as dementia and delirium may be secondary to the main presenting issue (e.g., urinary-tract infection, heart failure, etc.) and so may be under-reported [19]. Indeed, delirium can be a presenting symptom of COVID-19 in older adults [47]. Secondly, our data collection period concluded in October at the end of the second lockdown in Metropolitan Melbourne. Given the heightened risk of severe illness in older people infected with COVID-19, it is possible that presentations to emergency departments for mental health in this age group remained low for the duration of the lockdowns and that the true impact of lockdowns and deferred care may be revealed in subsequent time points. Evidence of this effect is indicated by ABS data on excess deaths, which suggest a 17.2% increase in deaths due to dementia in 2021 compared to the 2015–19 average, 5.7% higher than in 2020 [48].

Reasons for the decrease in developmental/behavioural disorder presentations remain unclear. This may indicate unexpected benefits of the pandemic for some groups, such as reduced pressure to engage in social settings leading to increased stability. Some findings of this effect have been observed in children and adolescents [49]. However, it is unclear if this applies to adults, and whether such benefits outweigh the negative impacts, including reduced access to services [49]. Further, it is possible that these changes could represent hesitancy to seek treatment due to misunderstanding of stay-at-home rules or fear of COVID-19 infection risk.

Future qualitative research should investigate the reasons for the change in ED presentations, especially for eating disorders and developmental/behavioural disorders, and clarify the impacts of deferred care and lockdowns on older adults with mental health conditions. Further research is required to understand whether adults with developmental/behavioural disorders have benefited from the reprieve in social life brought on by the pandemic, or whether the reduced presentations can be attributed to other causes that may exacerbate future illness.

## Strengths and Limitations

This study has several strengths. Firstly, we analysed a whole-of-state database with data disaggregated by mental health condition, age group, sex, SES, and metro/regional location, providing more nuanced data on the association between COVID-19 restrictions and mental health presentations. In comparison to some prior studies [13, 15], we analysed data over a three-year period, providing greater certainty in our predicted versus observed results. Our study also has limitations. Our design is retrospective, cross-sectional and our observation time is limited to the first eight months of the pandemic in Australia. Use of ICD-10-AM codes to classify mental health disorders may underestimate ED presentations for mental health, as ED clinicians typically only record one condition per presentation [19]. Our study may be limited by inaccuracies in diagnostic coding, as the ED diagnoses are generally entered by busy clinicians with limited formal training in coding [50]. The compressed list of ICD-10-AM codes utilised in the VEMD make disaggregation of some presenting issues impossible, such as suicide attempts and non-suicidal self-injury which both fall under the VEMD category of ‘self-harm’ [19]. Administrative data cannot elucidate why adults were more likely to present. Finally, our analysis cannot account for unmeasured exposures that may affect adult mental health, meaning our data can confirm associations but not infer causality.

## Conclusions

COVID-19 restrictions have seen some increases in adult presentations for mental health issues in metropolitan Victoria, particularly in young females. There were large increases in eating disorder presentations among young adults living in higher SES/less deprived areas. Consumers, clinicians, community health providers, and policy makers must work together to strengthen support for vulnerable young adults and women, including rapid upskilling of the existing workforces and longer-term funding of support that is accessible to those who need it. Further research is needed to understand the impact of delayed care on older adults with mental health issues, particularly those with cognitive impairment.

## Data Availability

The data that support this study were obtained from The Centre for Victorian Data Linkage (CVDL) by permission/licence. Data will be shared upon reasonable request to the corresponding author with permission from The Centre for Victorian Data Linkage (CVDL).

## Appendix. ICD-10 AM Codes for each Disorder Group

**Table:**

**Disorder group**	**ICD-10AM codes**
Dementia	F00-F03
Delirium	F05
Alcohol related disorders	F10
Substance abuse	F11-F19
Schizophrenia/delusional disorders	F20-F29
Mood disorders	F30-F39
Anxiety disorders	F40-F49
Eating disorders	F40-F49
Personality disorders	F60-F69
Self-harm	X60-X84 and S or T codes with intent of self-harm
Development/behavioural disorders	F70-F99
